# Purification, characterization and partial amino acid sequences of thermo-alkali-stable and mercury ion-tolerant xylanase from *Thermomyces dupontii* KKU–CLD–E2–3

**DOI:** 10.1038/s41598-020-78670-y

**Published:** 2020-12-10

**Authors:** Wasan Seemakram, Santhaya Boonrung, Tadanori Aimi, Jindarat Ekprasert, Saisamorn Lumyong, Sophon Boonlue

**Affiliations:** 1 Graduate School, Khon Kaen University, Khon Kaen, 40002 Thailand; 2 Biology Program, Faculty of Science, Buriram Rajabhat University, Buriram, 31000 Thailand; 3 Department of Biochemistry and Biotechnology, Faculty of Agriculture, Tottori University, Tottori, 680-8553 Japan; 4 Department of Microbiology, Faculty of Science, Khon Kaen University, Khon Kaen, 40002 Thailand; 5 Protein and Proteomics Research Center for Commercial and Industrial Purposes (ProCCI), Faculty of Science, Khon Kaen University, Khon Kaen, 40002 Thailand; 6 Department of Biology, Faculty of Science, Chiang Mai Univertity, Chiang Mai, 50200 Thailand; 7 Research Center of Microbial Diversity and Sustainable Utilization, Chiang Mai University, Chiang Mai, 50200 Thailand; 8 Academy of Science, The Royal Society of Thailand, Bangkok, 10300 Thailand

**Keywords:** Biochemistry, Biotechnology, Microbiology

## Abstract

We investigated the properties of the low molecular weight thermo-alkali-stable and mercury ion-tolerant xylanase production from *Thermomyces dupontii* KKU-CLD-E2-3. The xylanase was purified to homogeneity by ammonium sulfate, Sephadex G–100 and DEAE–cellulose column chromatography which resulted 27.92-fold purification specific activity of 56.19 U/mg protein and a recovery yield of 2.01%. The purified xylanase showed a molecular weight of 25 kDa by SDS–PAGE and the partial peptide sequence showed maximum sequence homology to the endo-1,4-β-xylanase. The optimum temperature and pH for its activity were 80 °C and pH 9.0, respectively. Furthermore, the purified xylanase can maintain more than 75% of the original activity in pH range of 7.0–10.0 after incubation at 4 °C for 24 h, and can still maintain more than 70% of original activity after incubating at 70 °C for 90 min. Our purified xylanase was activated by Cu^2+^ and Hg^2+^ up to 277% and 235% of initial activity, respectively but inhibited by Co^2+^, Ag^+^ and SDS at a concentration of 5 mM. The *K*_*m*_ and *V*_*ma*x_ values of beechwood xylan were 3.38 mg/mL and 625 µmol/min/mg, respectively. Furthermore, our xylanase had activity specifically to xylan-containing substrates and hydrolyzed beechwood xylan, and the end products mainly were xylotetraose and xylobiose. The results suggested that our purified xylanase has potential to use for pulp bleaching in the pulp and paper industry.

## Introduction

Xylan is the main carbohydrate found in hemicellulose consists of β-1,4-linked d-xylopyranose units for the main chain of a homo polymeric backbone, and short chain branches consisting of *O*-acetyl, α-L-arabinofuranosyl and α-D-glucuronyl residues^1,2^. Its complete hydrolysis requires the action of several different enzymes including endo-β-1,4-xylanase (EC 3.2.1.8) and β-xylosidase (EC 3.2.1.37) which released xylooligosaccharides and d-xylose as the main products, respectively^3,4^.

Xylanase has been widely used for application in several industries such as biorefinery, food, animal feed, pulp and paper, and textile industries. For the purpose of pulping and bleaching processes in pulp and paper industries, the xylanase should have high catalytic efficiency and must be stable and active both at high temperature and in alkaline condition, as well as tolerant to metal ions^5,6^. In addition, there is evidence that low molecular weight xylanase can effectively diffuse into the fibrous pulp and thus can efficiently hydrolyze xylan in biomass hydrolysis or pulp bleaching^7^. However, using xylanase in pulp bleaching process, there are still several properties needed to be considered. For example, enzyme should be free of cellulase and active at high temperature of, e.g., 50–90 °C and in alkaline pH of 8–10 for 30–300 min^8^. Recently, low-molecular-weight and thermo-alkali-stable xylanase have been observed in some species of thermophilic fungi. The thermophilic fungi are a good producer of extracellular xylanase which are thermostable, broad tolerance to pH variation, great resistance to denaturing agents, and have optimum activity at elevated temperatures^9–11^. However, the xylanase activity of these fungi was usually inhibited by metal ions, especially the mercury ion, Hg^2+^^12–15^. Therefore, it is important to search for new sources of xylanases production that have the required properties suitable for application in industry.

Previously, we reported the optimization of culture condition for the production of cellulase-free xylanase from the fungus, *T. dupontii* KKU–CLD–E2–3, whose crude xylanase activity was outstanding under alkaline and thermostable conditions^16^. These remarkable properties were interesting for application in pulp bleaching of paper industry. In order to achieve the pulp bleaching step, which is planned for the future, the xylanase produced by *T. dupontii* KKU–CLD–E2–3 was, therefore, purified and characterized. The partial sequence of amino acid consensus with endo-1,4-β-xylanase was also investigated in this present work. In addition, we are the first to report that the xylanase activity from this fungus was significantly activated by mercury ion (Hg^2+^). This novel finding suggested that our xylanase has a unique characteristic when compared to those of other xylanase-producing fungi whose activities are strongly inhibited by Hg^2+^.

## Materials and methods

### Materials

Beechwood xylan was purchased from Sigma Chemical Co., St. Louis, MO, USA. Sephadex G–100 gel filtration media was obtained from GE Healthcare in Uppsala, Sweden. Diethylaminoethyl (DEAE–cellulose) was purchased from Sigma-Aldrich. in St. Louis, MO, USA. AmershamTM ECLTM gel 4–20% Kit was purchased from Merck, Darmstadt, Germany (Silica Gel 60 F254). Xylose and xylooligosaccharide were purchased from Sigma Chemical (Wako Pure Chemical Industries. Ltd, USA.). All other chemicals were of analytical grade.

### The thermophilic fungi

*Thermomyces dupontii* KKU–CLD–E2–3 (Accession number LC428093) was used as the inoculums and was isolated from elephant dung collected from Chulabhorn Dam, Chaiyaphum province, Thailand^16^. This fungus was obtained from the Mycorrhizal and Fungal Technology Laboratory, Department of Microbiology, Faculty of Science, Khon Kaen University, Thailand.

### Xylanase production in solid-state fermentation (SSF) and enzyme extraction

In this study, the xylanase production in solid-state fermentation by the fungus *T. dupontii* KKU–CLD–E2–3 was performed by following the method described by Seemakram et al.^16^ in which fermentation conditions were optimized for the production process. The *T. dupontii* KKU–CLD–E2–3 was cultivated in 500 mL Erlenmeyer flask containing a 10 g of corn cob and 17 mL mineral solution (NH_4_Cl 1.77 g, KH_2_PO_4_ 3.0 g, MgSO_4_ 7H_2_O 0.5 g, CaCl_2_ 0.5 g, glucose 5.0 g per 1000 mL distilled water). The initial pH and moisture content of the medium was set to be 10.74 and 74.56%, respectively. Then, all flasks were sterilized at 121 °C for 15 min. Ten agar plugs (0.5 cm diameter) of a 4-d-old mycelial culture of the strain were inoculated in each flask, and incubated at 44.72 °C (Seemakram et al.^16^) under static conditions for 8 d. After that, 100 mL of 0.05 M Tris–HCl buffer having pH 9.0 was added to the cultures. We then shake the sample at 150 rpm and 30 °C for 60 min. The solid materials and fungal biomass were separated by centrifugation (13,551×*g*, 20 min). The supernatant was used for enzyme assays.

### Xylanase and protein assays

Xylanase activity was assayed by using beechwood xylan as substrate. A 0.5 mL of enzyme solution was added to 0.5 mL of 1% beechwood xylan solution in 0.05 M Tris–HCl buffer, pH 9.0. The mixture was incubated at 80 °C for 15 min, and the reducing sugar released was determined by Somogyi Nelson method^17^. One unit of xylanase activity was defined as the amount of enzyme required to liberate 1 μmol of reducing sugars from substrate per minute under the assay conditions. Protein was determined by the Lowry method^18^ using bovine serum albumin (BSA) as standard.

### Xylanase purification

The purification of crude xylanase was carried out with three steps consisted of ammonium sulfate precipitation, gel filtration and ion exchange chromatography by following the method described by Seemakram et al.^16^. The crude enzyme was precipitated by ammonium sulphate ((NH_4_)_2_ SO_4_) at concentration of 20–80% for overnight at 4 ºC. The precipitated protein was separated by centrifugation at 6000 rpm for 15 min and dissolve in 0.05 M Tris–HCl, pH 9.0, then precipitated protein was dialyzed against the same previously used buffer. Thereafter, 1.0 mL of protein sample was purified by gel filtration with Sephadex G–100 column (100 cm × 1.0 cm) and equilibrated with 0.05 M Tris–HCl buffer (pH 9.0). The protein sample was eluted by 0.05 M Tris–HCl buffer pH 9.0 at a flow rate of 0.25 mL/min, and 2 mL fractions were collected. Then, all active fractions were pooled and applied to final of purification step by ion exchange chromatography with a DEAE–Cellulose column (20 cm × 1.5 cm) equilibrated with the same previously used buffer at a flow rate of 0.5 mL/min. The bound proteins were eluted with a NaCl gradient (2 M) in the same buffer at a flow rate of 0.5 mL/min, and 2 mL fractions were collected. The highly active fractions were gathered and stored at − 40 °C for further characterization.

### SDS–polyacrylamide gel electrophoresis (SDS–PAGE)

The purified enzyme was checked for their purity by SDS–polyacrylamide gel electrophoresis (PAGE). The SDS–PAGE was performed following a modified method of Laemmli^19^ using AmershamTM ECLTM gel 4–20% Kit. Proteins in the gel were visualized by Coomassie brilliant blue R–250 staining. The chromatin pre-stained protein ladder (10–175 kDa) was used as a molecular weight marker.

### Protein identification by LC–MS/MS and data analysis

The protein was loaded in 14% acrylamide gel, and then visualized by Coomassie brilliant blue R–250. The specific band of target purified xylanase was cut by new clean scalpel blade and transferred into a microcentrifuge tube. The purified xylanase was analyzed for amino acid identification by LC–MS/MS system model API 2000 MDS SCIEX. The amino acid sequences were used for analyzing by the MASCOT program (www.matrixscience.com) and a database of annotated comparative protein structure models by the Protein Modeling 101 (https://www.proteinmodelportal.org) with the initial searching parameters; Enzyme: Trypsin; carbamidomethylation (C) as fixed modification, and oxidation (HW) and oxidation (M) as variable modification; peptide mass tolerance of 0.5 Da and fragment mass tolerance of 0.5 Da; a peptide charge state of + 1, + 2, + 3. The proteins were identified through the service of the Research Instrument Center, Khon Kaen University, Thailand.

### Effect of pH on the activity and stability of xylanase

Investigation for determining the optimum pH of purified xylanase activity was determined in a pH ranging from 3.0 to 12.0 at 80 °C and performed by following the method described by Seemakram et al.^16^. We used various buffers including 0.1 M McIlvaine (pH 3.0–8.0), 0.05 M Tris–HCl (pH 8.0–9.0), 0.05 M Glycine–NaOH (pH 9.0–10.0), and 0.05 M Na_2_HPO_4_-NaOH (pH 10.0–12.0). The pH stability of purified xylanase was pre-incubated in these buffers at 4 °C for 24 h. The residual activity was measured under the standard assay conditions.

### Effect of temperature on the activity and stability of xylanase

The optimum temperature for xylanase activity was determined at various temperatures (50–90 °C) in an optimum buffer and performed by following the method described by Seemakram et al.^16^. The thermal stability of purified xylanase in the optimum buffer was incubated at different temperatures of 50–90 °C for 90 min. After cooling, the residual xylanase activity was measured according to the standard assay conditions.

### Determination for substrate specificity and kinetic parameters

The purified xylanase was measured substrate specificity using 1% (w/v) of xylan consist of Beechwood xylan, Birchwood xylan, Oat spelt xylan, Carboxymethyl cellulose (CMC), Cellulose powder and Avicel, respectively^13^. The relative activity of enzyme for each substrate were analyzed at optimum temperature for 15 min and measured according to the standard assay conditions with slightly modification described by Seemakram et al.^15^. The kinetic experiments were performed at different concentrations of each substrate ranged from 2.5–30 mg/mL^14^ in an optimum buffer and incubated with the purified xylanase at optimum temperature for 15 min. The values of Michaelis–Menten constant (*K*_*m*_) and maximal reaction velocity (*V*_*max*_) were analyzed by the linear regression method describe by Lineweaver and Burk^20^.

### Effect of metal ions and reagents on xylanase activity

The purified xylanase was analyzed by pre-incubating the enzyme in solutions containing 1, 5 and 10 mM concentrations of different mineral salts (CuSO_4_, MgSO_4_, FeSO_4_, CoCl_2_, HgCl_2_, ZnCl_2_, AgNO_3_, MnSO_4_), ethylenediaminetetraacetic acid (EDTA) and sodium dodecyl sulfate (SDS) for 1 h at room temperature (25 ± 2 °C). Then, xylanase activity was compared to the control without adding metal ions and nonmetal reagents. The residual activity was then measured under the standard assay conditions.

### Hydrolysis products of beechwood xylan

The hydrolysis products from purified xylanase were determined by incubating the beechwood xylan with purified enzyme under the optimum conditions assay for 12 h. The hydrolyzed products were measured at different time intervals using thin layer chromatography (TLC) on silica gel plates. Xylose and xylooligosaccharide were used as standards. The products on TLC plates were developed using methanol: glacial acetic acid: H_2_O (6 : 7 : 2, v/v/v) as the mobile phase which modified from the method described by Li et al.^21^. The hydrolysis products were detected by spraying with methanol/sulfuric acid mixture (95 : 5, v/v), followed by heating at 100 °C for 15 min.

## Results

### Purification of xylanase

**Figure 1:**
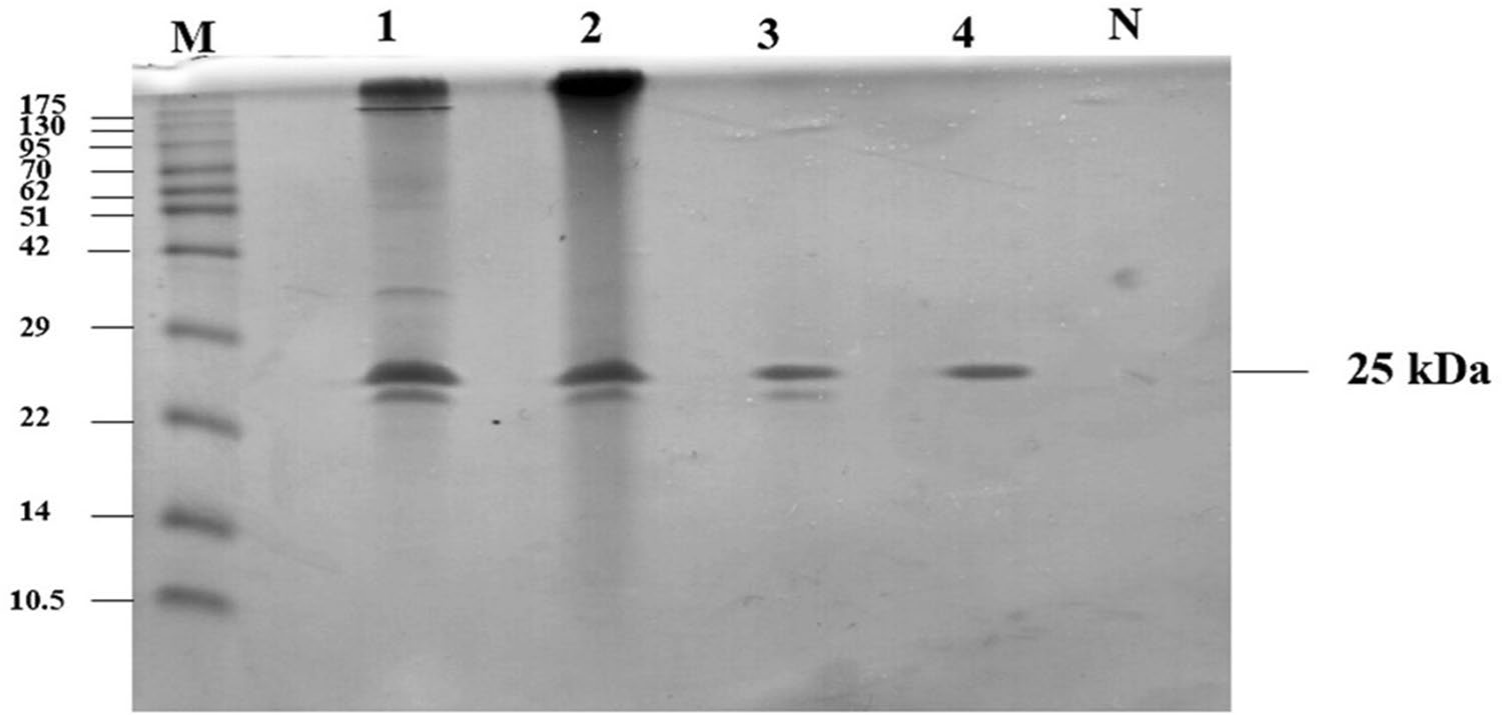
SDS–PAGE profiles of xylanase purification. Lane M: standard protein marker; 1: crude extract; 2: ammonium sulphate precipitation; 3: after gel filtration; 4: after DEAE-cellulose.

Purification of crude xylanase was achieved by classical method including ammonium sulfate precipitation, Sephadex G–100 gel filtration and DEAE–cellulose ion-exchange column chromatography. The crude xylanase, having a total activity of 5102.22 U and specific activity of 2.01 U/mg protein, was precipitated using ammonium sulfate to 30% saturation, further purified by Sephadex G–100 gel filtration and DEAE–cellulose ion-exchange column chromatography. After three steps of purification, the specific activity of the crude enzyme increased from 2.01 to 56.19 U/mg protein with a recovery yield of 2.01% and the 27.92-fold apparent homogeneity (Table 1). In addition, the purified xylanase from *T. dupontii* KKU–CLD–E2–3 was subjected to determination for molecular weight and homogeneous by SDS–PAGE. The purified xylanase appeared as a single protein band with a molecular mass of approximately 25 kDa (Fig. 1).

**Table 1:** Summary of the purification of xylanase from *Thermomyces dupontii* KKU–CLD–E2–3.

Purification steps	Total activity (Unit)	Total protein (mg)	Specific activity (U/mg)	Purification (fold)	Yield (%)
Crude enzyme	5102.22	2535.65	2.01	1.00	100.00
80% (NH_4_)_2_ SO_4_	508.80	32.32	15.74	7.82	9.97
Sephadex G–100	103.06	2.22	46.48	23.10	2.02
DEAE–cellulose	102.60	1.83	56.19	27.92	2.01

### Protein identification by LC–MS/MS and data analysis

The sample from *T. dupontii* KKU–CLD–E2–3 was analyzed with amino acid identification of purified analyze by LC–MS/MS. The obtained peptide sequences were used for analyzing by the MASCOT search (Table 2), which amino acid similar to those fungi previously reported (Table 3). This strain was related to members of the description endo-1,4-β-xylanase, where it showed maximum sequence identities of 90% and similarity of 1.452 at resolution 1.55 Å with the endo-1,4-β-xylanase from *Thermomyces lanuginosus* (Accession number gi|3915307).

**Table 2:** Protein sequence of purified xylanase from *Thermomyces dupontii* KKU–CLD–E2–3. Protein sequence coverage of 31% indicated identity or extensive homology (*P* < 0.05).

Start–End	Observed ion (m/z)		Expected molecular mass	Calculated molecular mass	Peptide		Icon score
81–89	492.7240		983.4334	983.4937	K. GWNPGLNAR.A		25
154–172	728.6470		2182.9192	2183.0284	R. VNAPSIDGTQTFDQYWSVR.Q		74
177–192	598.5740		1792.7002	1792.795	R. TSGTVQTGCHFDAWAR.A		45
193–218	951.3950		2851.1632	2851.3202	R. AGLNVNGDHYYQIVATEGYFSSGYAR.I		21
	1	MVGFTPVALA	ALAATGALAF	PAGNATELEK	RQTTPNSEGW	HDGYYYSWWS	
	51	DGGAQATYTN	LEGGTYEISW	GDGGNLVGGK	**GWNPGLNAR**A	IHFEGVYQPN	
	101	GNSYLAVYGW	TRNPLVEYYI	VENFGTYDPS	SGATDLGTVE	CDGSIYRLGK	
	151	TTR**VNAPSID**	**GTQTFDQYWS**	**VR**QDKR**TSGT**	**VQTGCHFDAW**	**ARAGLNVNGD**	
	201	**HYYQIVATEG**	**YFSSGYAR**IT	VADVG			

Bold letters show the matched peptides and sequence coverage found by MS/MS analysis against hypothetical GH 11 xylanase (Model ID O43097, Accession number gi|3915307).

**Table 3:** Identification of purified xylanase from *Thermomyces dupontii* KKU–CLD–E2–3.

NCBInr ID	Nominal mass (Mr)	Sequencing of generated peptide by MS/MS	Protein identification	Organism
This work	2851	WNPGLNARVNAPSIDG-T-QTFDQYWSVRT–––-SG–-TVQ–––––TGCHFDAWA—RAGLNVNGDHY––––YQ IVATE-Y–-F—SSGYAR	Endo-1,4-β-xylanase	*Thermomyces dupontii* KKU–CLD–E2–3
gi|3915307	24,455	YRLGKTTRVNAPSIDG-T-QTFDQYWSVRQDKRT–-SG–-TVQ–––––TGCHFDAWA—RAGLNVNGDHY––––YQIV ATEGY–-F–SSGYA	Endo-1,4-β-xylanase	*Thermomyces lanuginosus* $1YNA

### Effect of pH on the activity and stability of xylanase

**Figure 2:**
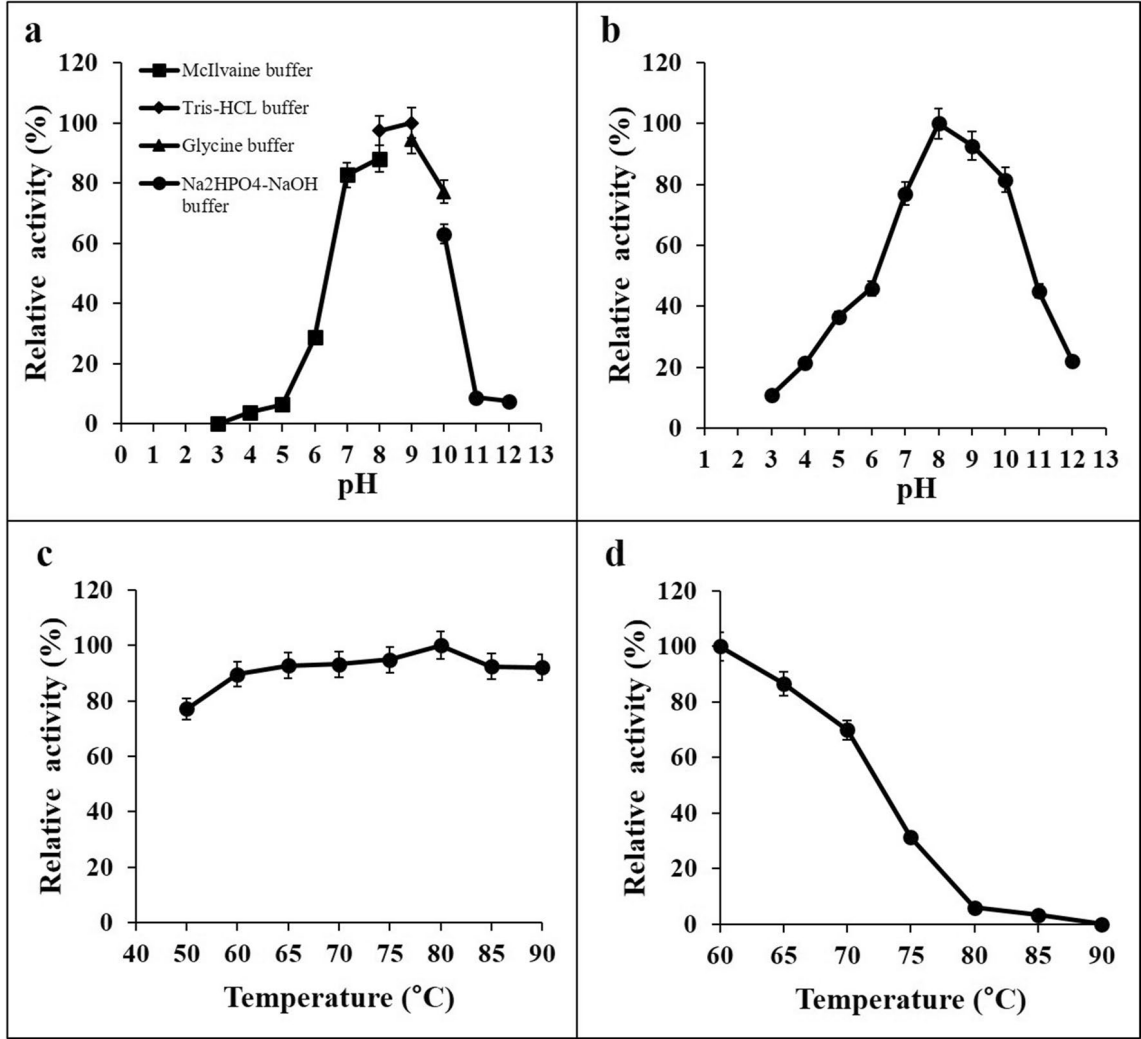
Effect of the optimum pH (**a**), the pH stability (**b**), the optimum temperature (**c**) and the thermal stability (**d**) of the endo-1,4-β-xylanase of *Thermomyces dupontii* KKU–CLD–E2–3.

The purified endo-1,4-β-xylanase from *T. dupontii* KKU–CLD–E2–3 was active at pH 9.0 (Fig. 2a), a higher pH than those described in previous reports for other fungi (Table 4). The purified endo-1,4-β-xylanase retained over 75% of the original activity in pH range of 7.0–10.0 after incubation at 4 °C for 24 h (Fig. 2b).

**Table 4:** Some enzymatic properties of purified xylanase from xylanase-producing thermophilic fungi.

Fungi	Mw (kDa)	Optimum pH	Optimum temperature	pH stabilily	Thermostability	References
*Thermomyces dupontii* KKU–CLD–E2–3	25	9.0	80 °C^a^	7.0–10.0^a^	> 70%, 70 °C^b^	This work
*Thermomyces lanuginosus* CSB 288.54	26.2	7.0–75	70 °C	6.5–10.0	50%, 85 °C	Li et al.^5^
*Thermomyces aurantiacus* var*. levisporus* KKU–PN–I2–1	27	9.0	60 °C	7.0–9.0	70%, 50 °C	Chanwicha et al.^13^
*Myceliophthora thermophila* BF1–7	14	12	50 °C	9.0–12.0	56%, 50 °C	Boonrung et al.^14^
*Talaromyces thermophilus* F1208	21.6	6.5	55 °C	5.5–6.5	55%, 50 °C	Li. et al.^31^
*Myceliophthora heterothallica* F.2.1.4	NR^c^	6.0	55 °C	4.5–9.5	50%, 65 °C	Ping et al.^32^
*Sporotrichum thermophile*	24	5.0	60 °C	4.0–8.0	90%, 50 °C	Vafiadi et al.^34^
*Thermomyces lanuginosus* 195	22	8.0	50 °C	5.0–10.0	87%, 70 °C	Gaffney et al.^42^
*Myceliophthora thermophila*	25, 27	6.0	60 °C	6.0–8.0	60%, 60 °C	Yegin^43^
*Thermoascus aurantiacus* M–2	31	5.0	75 °C	2.0–10.0	70%, 70 °C	Basit et al.^44^

^a^Pre-incubation at 4 °C, 24 h.

^b^Pre-incubation at pH 9.0, 90 min.

^c^Not reports.

### Effect of temperature on the activity and stability of xylanase

The endo-1,4-β-xylanase from *T. dupontii* KKU-CLD-E2-3 was optimally active at 80 °C (Fig. 2c). For thermo-stability, xylanase activity was retained more than 70% of the original activity after heating at 70 °C for 90 min (Fig. 2d).

### Substrate specificity and kinetic constants

The substrate specificity of the purified endo-1,4-β-xylanase was determined on several substrates (Table 5). The results showed that the highest activity was obtained in beechwood xylan, followed by birchwood xylan, and oat spelt xylan, respectively. In contrast, the xylanase did not have an activity towards Avicel, CMC and cellulose powder. The *K*_*m*_ and *V*_*max*_ of endo-1,4-β-xylanase was determined by beechwood xylan as a substrate. In this case, the *K*_*m*_ and *V*_*max*_ values of the purified endo-1,4-β-xylanase were 3.38 mg/mL and 625 µmol/min/mg, respectively.

**Table 5:** Substrate specificity of the purified xylanase from *Thermomyces dupontii* KKU–CLD–E2–3.

Substrate (1% w/v)	Relative activity (%)
Beechwood xylan	100.00 ± 0.10
Birchwood xylan	18.23 ± 0.30
Oat spelt xylan	3.88 ± 0.10
Avicel	0.00 ± 0.00
CMC	0.00 ± 0.00
Cellulose powder	0.00 ± 0.00

### Effect of metal ions and reagents on xylanase activity

The effect of different metal ions and reagents on the activity of the purified xylanase from *T. dupontii* KKU–CLD–E2–3 is summarized in Table 6. The addition of metal ions and nonmetal reagents at a concentration of 1 mM showed increased xylanase activity, except for Ag^+^ and SDS. The presence of Cu^2+^, Mg^2+^, Fe^2+^, Hg^2+^, Zn^2+^, Mn^2+^ and EDTA at a concentration of 5 mM increased xylanase activity, especially Cu^2+^ and Hg^2+^ that increased the xylanase activity up to 277% and 235%, respectively. On the other hand, Co^2+^, Ag^+^ and SDS at a concentration of 5 mM showed inhibition of enzyme activity. In addition, xylanase activity was significantly activated by Cu^2+^ and Mg^2+^ at a concentration of 10 mM up to 248% and 259% of activity, respectively.

**Table 6:** Effect of different metal ion and reagents on xylanase activity from *Thermomyces dupontii* KKU–CLD–E2–3.

Metal ions/reagent	Relative activity (%)		
	1 mM	5 mM	10 mM
Non	100.00 ± 0.00	100.00 ± 0.00	100.00 ± 0.00
CuSO_4_	122.90 ± 0.50	277.67 ± 0.26	248.24 ± 0.21
MgSO_4_	105.55 ± 0.47	117.81 ± 0.17	259.57 ± 0.05
FeSO_4_	137.94 ± 0.31	135.16 ± 0.28	64.14 ± 0.30
CoCl_2_	185.31 ± 3.90	92.60 ± 0.25	27.47 ± 0.37
HgCl_2_	180.22 ± 0.20	235.22 ± 0.23	54.08 ± 0.77
ZnCl_2_	115.73 ± 0.13	131.06 ± 0.86	64.89 ± 0.42
AgNO_3_	79.01 ± 0.13	88.55 ± 0.18	85.48 ± 0.04
MnSO_4_	121.40 ± 0.10	139.33 ± 0.39	35.22 ± 0.01
EDTA	123.13 ± 0.19	112.84 ± 1.09	60.79 ± 0.76
SDS	72.41 ± 0.02	65.30 ± 0.01	50.90 ± 0.20

### Hydrolysis properties of the purified xylanase

**Figure 3:**
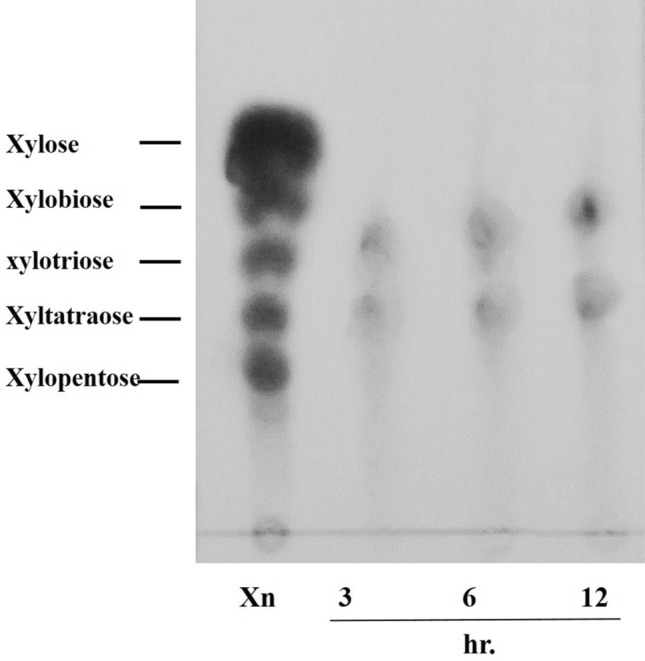
TLC analysis of hydrolysis of beechwood xylan by the endo-1,4-β-xylanase from *T. dupontii* KKU–CLD–E2–3: standard xylose and xylooligosaccharide marker (Xn) and time of incubation (h).

The hydrolysis of beechwood xylan by purified endo-1,4-β-xylanase was studied using thin layer chromatography (TLC) (Fig. 3). The result revealed that the purified endo-1,4-β-xylanase liberated mainly xylotriose and xylotetraose from beechwood xylan.

## Discussion

Among xylanase-producing thermophilic fungi, there are only a few reports on the properties of xylanase from *Thermomyces dupontii*. In this research, the thermo-alkali-stable and metal ions-tolerant xylanase from *T. dupontii* KKU–CLD–E2–3 was purified and characterized. The purification fold of xylanase purification in this study was higher than some previous studies using other thermophilic fungi. For instance, the observations of Kumar and Shukla^22^ showed that the xylanase from *Thermomyces lanuginosus* VAPS24 was purified 5.92-fold using acetone precipitation followed by ultrafiltration. According to Yang et al.^23^, the purified xylanase from *Aspergillus fumigatus* Yang FC2–2 was purified to 25.5-fold and apparent homogeneity of 13.9% recovery yield. Maalej et al.^24^ reported that the purified xylanase from *Talaromyces thermophilus* was purified 23-fold using diethylaminoethyl cellulose anion exchange chromatography, P–100 gel filtration, and Mono Q chromatography. Chanwicha et al.^13^ also reported purification of xylanase to 14.5-fold with 2.3% recovery from *Thermoascus aurantiacus* var. *evisporus* KKU–PN–I2–1. The purified xylanase from *T. dupontii* KKU–CLD–E2–3 was appeared as a single protein band on SDS-PAGE gel with a molecular mass of approximately 25 kDa, which was in agreement with the value of 25 kDa xylanase from *T. thermophiles*^24^ and 25.8 kDa xylanase from *Paecilomyces thermophila* J18^10^. The apparent molecular mass of the xylanase in this study was similar to the 26.2 kDa xylanase from *Thermomyces lanuginosus* CBS 288.54^5^ and 27 kDa xylanase from *T. aurantiacus* var. *levisporus* KKU–PN–2–1^13^.

Then, this protein band was identified against genome sequence assembly v1.0 database (MASCOT search) and the Protein Modeling 101 in order to obtain peptide sequences. The bands excised from the gel were analyzed by mass spectrometry and were compared to protein sequences available in the NCBI database revealed. The protein sequence matches with Endo-1,4-β-xylanase of *Thermomyces* spp. at 90% identity (Accession number: gi|3,915,307)^25^. In addition, these results showed that identities were similar to protein from *Thermomyces lanuginosus* (Accession number:gi|335,371,365) at 89% identity^26^. The relatively high yield of enzyme and the coverage of peptides from mass-spec was so sparse which the protein sequence coverage of 31% indicated that identity or extensive homology from mass-spectrometry has reliability. Because of the individual ions scores > 54 of indicating identity have the significance at p < 0.05 of mascot score histogram. Besides, the peptide sequence of xylanase has the glycans attached in the fragment after cutting with the specific enzyme^27^ that attached to the amide group of an asparagine residue within the consensus peptide sequence^28^, resulting in the observed ion (m/z) to high value has changed it is not matches the coverage of another peptide sequence in the database. The results of the experiments in Table 5 confirmed that this enzyme was xylanase.

According to previous literature, the purified xylanase from *Humicola grisea* var. *thermoidea* was most active at pH of 4.5–6.5^29^. Maalej et al.^24^ reported that the optimum pH of the purified xylanase from *T. thermophilus* was 7.0. The optimum pH of xylanase from *Thermomyces lanuginosus* THKU–49 was 6.0^30^. Furthermore, Li et al.^31^ found that the hybrid xylanase enzyme (TXynFM) from *Talaromyces thermophilus* F1208 had optimum activity at pH 6.5. Also, Ping et al.^32^ reported that the purified xylanase from *Thermoascus aurantiacus* M–2 was most active at pH 5.0. In addition, the highest xylanase activity from *Myceliophthora heterothallica* F.2.1.4 was observed at pH 6.0^33^. Our results showed that the purified xylanase from *T. dupontii* KKU–CLD–E2–3 was most active at pH 9.0, which was higher pH than those reported for other thermophilic fungi. Because this fungus could be grown in alkaline pH conditions resulted in the enzyme production by this fungus had an optimal pH higher than the others fungi which similar to that from the report from Chanwicha et al.^13^.

The purified xylanase from strain KKU–CLD–E2–3 remained more than 75% of the original activity in pH range 7.0–10.0 after incubation at 4 °C for 24 h. This was in contrast to Vafiadi et al.^34^ who found that the hybrid xylanase from *Sporotrichum thermophile* was stable in a pH range of 4.0–8.0. The purified xylanase from *T. lanuginosus* THKU–49 was stable at pH 3.5–8.0^30^. In addition, Amo et al.^35^ found that the purified xylanase from *M. heterothallica* F.2.1.4 was stable in pH range of 4.5–9.5.

In terms of optimum temperature of enzyme, the optimum temperature of the purified enzyme from strain KKU–CLD–E2–3 was found to be 80 °C. Interestingly, the optimum temperature of this xylanase was higher than those from the other xylanase-producing thermophilic fungi. Li et al.^5^ reported that xylanase from *T. lanuginosus* CBS 288.54 was optimally active at 70–75 °C, while Maalej et al.^24^ reported the optimum temperature of the purified xylanase from *T. thermophiles* to be 75 °C. Khucharoenphaisan et al.^30^ found that the optimum pH of xylanase from *Thermomyces lanuginosus* THKU–49 was 70 °C. Also, Chanwicha et al.^13^ reported that the purified xylanase from *T. aurantiacus* var. *levisporus* KKU–PN–I2–1 was optimally active at 60 °C. The purified xylanase from the thermophilic fungus *M. thermophila* BF1–7 was 50 °C^14^. In addition, the maximum activity of purified xylanase from *T. aurantiacus* M–2 was determined at 75 °C^32^. As for the thermo-stability of enzyme from our strain KKU–CLD–E2–3, xylanase activity was retained more than 70% of the original activity after heating at 70 °C for 90 min. This result was similar to values for the xylanase from other strains of thermophilic fungi^13,30,32^.

The highest hydrolytic activity of purified xylanase was exhibited toward beechwood xylan, followed by birchwood xylan and oat spelt xylan, respectively. Our results indicated that substrate specificity of xylanase depends on type of xylan substrates. The purified xylanase in this study did not act towards Avicel, carboxymethyl cellulose and cellulose powder. These results indicated that the purified xylanase from *T. dupontii* KKU–CLD–E2–3 was a true xylanase and belongs to the glycosylic family G11. The xylanases of family G11 have no activity on cellulose and are the low-molecular-weight enzymes^24^. The *K*_*m*_ and *V*_*max*_ values of the purified xylanase in this study were 3.38 mg/mL and 625.00 µmol/min/mg, respectively. This study then provided better value of both *K*_*m*_ and *V*_*max*_ in comparison to the value previously reported from *T. thermophilus* (*K*_*m*_ 22.51 mol/L and *V*_*max*_ 1.235 µmol/min/mg)^24^, *T. aurantiacus* var. *levisporus* KKU-PN-I2-1 (*K*_*m*_ 40.9 mol/L and *V*_*max*_ 6.2 µmol/min/mg)^13^, *M. thermophila* BF1-7 (*K*_*m*_ 9.67 mol/L and *V*_*max*_ 5.38 µmol/min/mg)^14^, and *T. aurantiacus* M-2 (*K*_*m*_ 4.81 mol/L and *V*_*max*_ 467.2 µmol/min/mg)^32^.

The purified xylanase activity in this study was stimulated up to 277% and 235%, respectively by Cu^2+^ and Hg^2+^ but inhibited by Co^2+^, Ag^+^ and SDS at a concentration of 5 mM. In addition, xylanase activity was significantly activated by Cu^2+^ and Mg^2+^ at a concentration of 10 mM up to 248% and 259%, respectively. According to Boonrung et al.^14^, the purified xylanase from *M. thermophila* BF1–7 was activated by Cu^2+^, Mg^2+^ and Ag^+^ at 1 mM. The observations of Chanwicha et al. ^13^ showed that the xylanase from *T. aurantiacus* var. *levisporus* KKU–PN–I2–1 was stimulated by Cu^2+^and Mg^2+^ but was inhibited by Ag^+^ at 1 mM. Maalej et al.^24^ reported that the purified xylanase from *T. thermophilus* was stimulated by Ag^+^ but was inhibited by Mg^2+^ at 10 mM. Also, Kumar and Shukla^22^ found that the xylanase from *T. lanuginosus* VAPS24 was strongly inhibited by Cu^2+^ at a concentration of 10 mM. While the Hg^2+^ has previously been reported to completely inhibit the activity of xylanase^13,14,22,24,35,36^ it should be noted that our purified xylanase showed good stability in the presence of Hg^2+^. The active effect of Hg^2+^ ions is possibly due to its binding to the free SH group of thiol acid, the interaction of carboxyl group and/or the imidazole group of amino acid^37^, which the thiol groups are essential for enzyme activity, because thiol groups are one of the main targets of heavy metals. However, HgCl_2_ is a known inhibitor of thermitase through its binding to the free SH group^38^. Besides, the effects of metal ions and reagent to enzyme inhibition such as Co^2+^ and Ag^2+^ are mixed inhibitors, which do not bind to the active site, but to another region of the enzyme, and thus to the formation of complex with the reactive groups of the enzyme, resulted in reducing of the enzyme availability function^39^. In addition, the enzyme activity is inhibited by SDS, which interferes in hydrophobic regions of the enzyme, resulted in alternate of its three-dimensional structure indicating that cause enzyme denaturation^40^.

The purified xylanase from *T. dupontii* KKU–CLD–E2–3 hydrolyzed beechwood xylan to yield mainly xylotetraose, xylotriose and xylose as end products. This result indicated that the purified xylanase of this fungus was endo-xylanase. These data were in agreement with the observations of Li et al.^29^ which found that the xylanase from *Talaromyces thermophilus* F1208 mainly liberated xylotriose, xylotetraose and xylopentaose from beechwood xylan as substrates. The purified xylanase from *Aspergillus carneus* M34 mainly liberated xylotriose and xylotetraose from beechwood xylan^41^.

## Conclusions

In this study, we firstly reported the amino acid sequence of the low-molecular-weight thermo-alkali-stable xylanase of *T. dupontii* KKU–CLD–E2–3. Until now, no previous studies have been reported on the mercury ion (Hg^2+^) tolerant xylanase from thermophilic fungi. Interestingly, our purified xylanase was resistant to HgCl_2_ (Hg^2+^). In addition, these enzyme characteristics suggested the high potential in the pulp and paper industry. Since the optimum pH and the temperature of purified xylanase activity are relatively similar to the pH of the initial pulp and the temperature of bleaching activity in the factory processes. The enzyme was used in bleaching processes without pH and temperature alteration in the standard bleaching process of the factory production line. In addition, purified xylanase had high specific activity and low molecular weight, which ease to access into the fiber wall structure and can efficiently hydrolyze xylan in pulp bleaching without adverted effect on the structure of cellulose in pulp fiber, resulting in an improvement of pulp and paper quality. Therefore, this enzyme can be used in place of chemicals in bleaching process, which more environmentally-friendly than the chemical bleaching. To verify the potential use of purified xylanase from *T. dupontii* KKU–CLD–E2–3 in bleaching step, further experiment is needed to be carried out under the actual condition, which is planned for the future.
